# Stronger enhancer II/core promoter activities of hepatitis B virus isolates of B2 subgenotype than those of C2 subgenotype

**DOI:** 10.1038/srep30374

**Published:** 2016-07-27

**Authors:** Yanli Qin, Xueshi Zhou, Haodi Jia, Chaoyang Chen, Weifeng Zhao, Jiming Zhang, Shuping Tong

**Affiliations:** 1 Department of Infectious Diseases, Huashan Hospital, Fudan University, Shanghai, China; 2 Key Lab of Medical Molecular Virology, School of Basic Medical Sciences, Fudan University, Shanghai, China; 3 Department of Infectious Diseases, the First Affiliated Hospital of Soochow University, Suzhou, Jiangsu Province, China; 4 The Liver Research Center, Rhode Island Hospital, Warren Alpert School of Medicine, Brown University, Providence, RI, USA

## Abstract

Hepatitis B virus (HBV) genotype C causes prolonged chronic infection and increased risk for liver cancer than genotype B. Our previous work revealed lower replication capacity of wild-type genotype C2 than B2 isolates. HBV DNA replication is driven by pregenomic RNA, which is controlled by core promoter (CP) and further augmented by enhancer I (ENI) and enhancer II (ENII). DNA fragments covering these regulatory elements were amplified from B2 and C2 isolates to generate luciferase reporter constructs. As ENII is fully embedded in CP, we inserted HBV DNA fragments in the sense orientation to determine their combined activities, and in the antisense orientation to measure enhancer activities alone. Genotype B2 isolates displayed higher ENI+ENII+CP, ENII+CP, and ENII activities, but not ENI or ENI+ENII activity, than C2 isolates. The higher ENII+CP activity was partly attributable to 4 positions displaying genotype-specific variability. Exchanging CP region was sufficient to revert the replication phenotypes of several B2 and C2 clones tested. These results suggest that a weaker ENII and/or CP at least partly accounts for the lower replication capacities of wild-type C2 isolates, which could drive the subsequent acquisition of CP mutations. Such mutations increase genome replication and are implicated in liver cancer development.

Hepatitis B virus (HBV) isolates worldwide can be classified into eight genotypes (A-H) and further divided into subgenotypes^1^,^2^,^3^. Genotypes B and C co-circulate in East Asian countries such as China. Through their perinatal mode of transmission, these two genotypes are responsible for majority of chronic HBV infection worldwide. Chronically infected individuals are initially positive for hepatitis B e antigen (HBeAg), a secreted version of viral core (capsid) protein, in the bloodstream. Subsequent seroconversion (loss of HBeAg followed by rise of anti-HBe antibody) is often accompanied by a marked decline in viral load in the liver and bloodstream, which is attributed to immune-mediated clearance through both cytolytic- and noncytolytic mechanisms. However, the cytolytic mechanism is a double-edged sword, and increased hepatocyte turnover promotes the development of liver cirrhosis and hepatocellular carcinoma (HCC). Independent studies demonstrated that genotype C patients seroconvert from HBeAg to anti-HBe about 10 years later than genotype B patients^4^,^5^,^6^, and consequently the prolonged phase of active viral DNA replication and protein expression increases the lifelong risk for liver cirrhosis and HCC^7^,^8^,^9^,^10^. In addition, genotype C isolates respond to interferon therapy less favorably than genotype B isolates^11^,^12^, and are more likely implicated in breakthrough infection of newborns from HBeAg positive mothers despite combined active/passive immunization^13^. Moreover, adulthood infection with genotype C has greater risk to become chronic^14^. On the other hand, genotype B infection is associated with higher risk for fulminant hepatitis and acute exacerbation of chronic infection^15^,^16^,^17^. To better understand the contrasting clinical features between these two major HBV genotypes would require their comparative functional studies.

We previously initiated such a study with a focus on the B2 and C2 subgenotypes prevalent in China. The 3.2-kb full-length HBV genome was amplified from serum samples of chronically infected patients residing in China and US, respectively, and cloned to pUC18 vector. Since HBV DNA replication is driven by the 3.5-kb terminally redundant pregenomic RNA, the cloned genome pool was released from the vector by restriction enzyme digestion followed by re-circularization. Alternatively, the HBV genome cloned to pUC18 vector via the SphI site was converted to tandem dimer via the same site (SphI dimer). Transient transfection of such replication competent forms of HBV DNA into Huh7 cells, a human hepatoma cell line, revealed lower replication capacity of most C2 clones or isolates than B2 clones or isolates^18^. On the other hand, C2 clones or isolates showed more efficient virion secretion.

The aim of the present study was to clarify the molecular basis for differential replication capacities of the C2 vs. B2 subgenotypes. Since transcription of the pregenomic RNA is driven by the core promoter (CP) and further augmented by the two enhancer elements^19^,^20^,^21^, we used reporter assays to compare these transcriptional regulatory elements between isolates of the two subgenotypes. The element found to be more active in genotype B2 was exchanged between clones of the two subgenotypes so as to establish its relevance to the differential replication capacity.

## Materials and Methods

### Reporter constructs to measure enhancer and promoter activities

**Figure 1:**
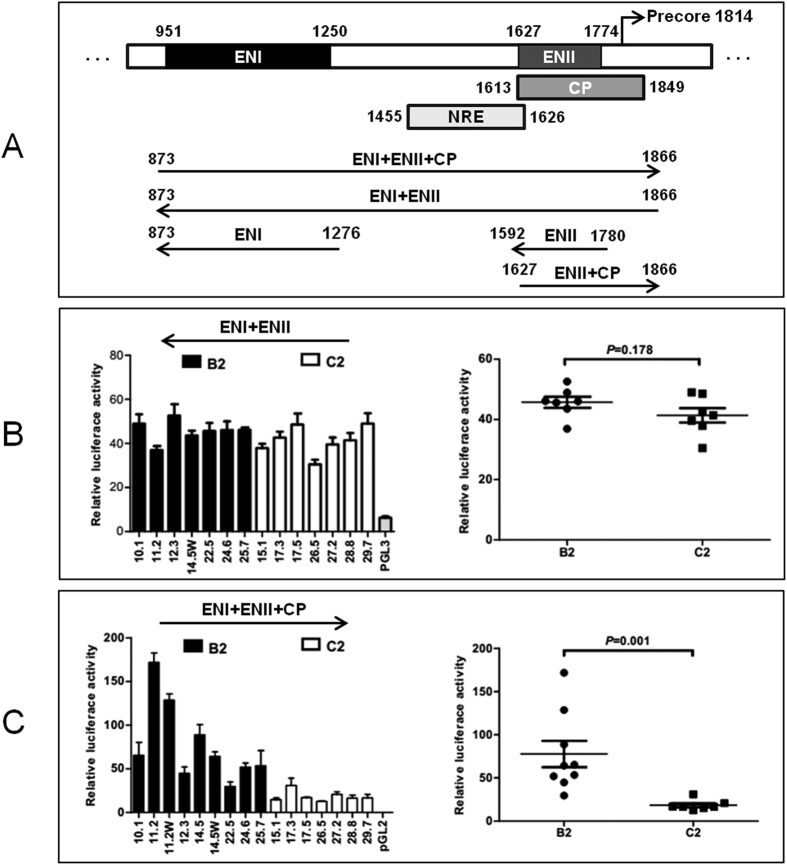
Schematic representation of the HBV transcriptional regulatory elements and luciferase reporter activities of a 1-kb HBV DNA fragment inserted in the sense or antisense orientation. (**A**) Location of ENI, NRE, ENII, and CP in the HBV genome. The location of NRE is based on ref. 31. Also shown are the reporter constructs used in the present study. Three HBV DNA fragments were inserted to pGL3 promoter vector in the antisense orientation to measure just enhancer activities, while two DNA fragments were inserted to pGL2 basic vector in the sense orientation to determine both enhancer and promoter activities. (**B**) Reporter activities of a 1-kb HBV DNA fragment (873–1866) inserted in the antisense orientation to measure combined effects of ENI+NRE+ENII. (**C**) Reporter activities of the same DNA fragment inserted in the sense orientation to reflect combined effects of ENI+NRE+ENII+CP. The relative luciferase levels (firefly luciferase activity/renilla luciferase activity) are shown as columns in the left panels and as dots in the right panels. The *P* values for the difference between the B2 and C2 isolates are provided. Data shown are based on three independent transfection experiments.

An HBV DNA fragment covering enhancer I (ENI), enhancer II (ENII) and CP (positions 873–1866; Fig. 1A) was amplified by polymerase chain reaction (PCR) from SphI dimers of HBV clones from U.S. patients^18^ (see Supplementary Table 1 for primer sequences), and subcloned to pGL2 Basic vector (Promega) in the sense orientation, or to pGL3 Promoter vector (Promega) in the antisense orientation. Similarly, a DNA fragment covering ENI (positions 873–1276) or ENII (positions 1592–1780) was subcloned to pGL3 Promoter vector in the antisense orientation, while a DNA fragment covering CP (1627–1866) as well as part of ENII was subcloned to the pGL2 Basic vector in the sense orientation. All constructs were verified by DNA sequencing and DNA was purified by HiSpeed Plasmid Midi Kit (Qiagen) for transfection experiments.

### Assays for promoter/enhancer activities

Huh7 cells were maintained in Dulbecco’s modification of Eagle’s medium (DMEM) supplemented with 10% fetal bovine serum. Cells seeded at 1–1.5 × 10^5^/wells in 24-well plates were co-transfected with 0.3 μg of HBV reporter construct expressing firefly luciferase and 6.25 ng of Rluc plasmid expressing renilla luciferase, using TransIT-LT1 reagent (Mirus). Activities of the two types of luciferase were measured from cell lysate two days later using Dual-Luciferase Reporter Assay System (Promega). The enhancer or promoter/enhancer activity was calculated as the ratio of firefly luciferase activity over renilla luciferase activity, and the results shown were based on three repeat experiments.

### Role of the ENII+CP overlapping region on HBV replication capacity

To exchange ENII+CP region (positions 1627–1866) or just its 5′ end (position 1630–1660) between SphI dimers of B2 and C2 subgenotypes, a chimeric DNA fragment covering positions 1571–2398 or 1571–2815 was generated by overlap extension PCR. The PCR product was digested with RsrII (position 1571) together with PshAI (position 2398) or BstEII (position 2815) for replacement of the cognate sequence in the original SphI dimer. Since the enzymatic manipulation converted the SphI dimer into a monomer, each chimeric construct was remade into a dimer using a newly developed method^22^. Huh7 cells grown in 6-well plates were transfected with SphI dimers, and harvested 3 days later. Southern blot analysis of replicative HBV DNA was performed according to our established protocol using mixed B2/C2 probes and washing at high salt (2XSSC) concentration^18^,^23^,^24^.

### Northern blot analysis

Detailed procedures have been described^18^,^23^,^24^. Briefly, Huh7 cells were lysed at day 3 post- transfection using TRIzol. RNA (15 μg) was denatured at 58 °C for 10 min and separated in agarose gel with morpholinepropanesulfonic acid and formaldehyde. After transfer, the Northern blots were hybridized with a ^32^P-labeled, mixed B2/C2 DNA probe. For loading control, the blots were stripped by boiling and reprobed with ^32^P-labeled glyceraldehyde 3-phosphate dehydrogenase (GAPDH) DNA.

### Western blot analysis

Core protein was detected by Western blot analysis using 1:2,000 dilution of a custom made rabbit antibody against core protein^25^ (a kind gift from Dr. Haitao Guo, Indiana University, USA), and 1:100,000 dilution of horse radish peroxidase (HRP)- conjugated anti-rabbit antibody. For loading control, the blots were incubated with 1:10,000 dilution of a mouse antibody against GAPDH, followed by anti-mouse secondary antibody.

### Statistical analysis

Transcription activities are expressed as mean ± SD. Differences of transcription activities between HBV genotype B and C were compared by Student’s *t* test or Wilcoxon Ranksum test. All tests were 2 sided. Statistical significance was defined as *P* < 0.05.

## Results

### Genotype C2 clones had weaker combined ENI+ENII+CP activities than genotype B2 clones

As shown in Fig. 1A, ENI, ENII, and CP are located at positions 951–1250, 1627–1774, and 1613–1849 of the HBV genome, respectively. Thus, ENII is embedded in CP, making it difficult to separate their activities. Considering that an enhancer, but not a promoter, can work in both sense and antisense orientations, we inserted relevant HBV DNA fragments to the pGL2 Basic vector in the sense orientation to quantify both enhancer and promoter activities, or to pGL3 Promoter vector in the antisense orientation to measure just the enhancer activity. As the current study was a follow up of our previous work on B2 and C2 clones with well defined biological properties^18^, these clones were employed for the PCR amplification of the transcriptional regulatory elements.

In the initial experiment, a 1-kb DNA fragment (873–1866) encompassing all the three transcriptional elements was cloned to the reporter plasmid in the sense orientation. The seven genotype C2 clones produced much lower promoter/enhancer activities (mean value: 18.41) in transiently transfected Huh7 cells than the nine B2 clones (mean value: 77.67) (*P* = 0.001) (Fig. 1C). For B2 clones 11.2 and 14.5 with naturally occurring CP mutations, back mutations reduced promoter activities (11.2 W and 14.5 W). Nevertheless, the difference between the two genotypes remained statistically significant even if these two clones were excluded from comparison (*P* = 0.003). When the same DNA fragment was subcloned to pGL3 Promoter vector in the antisense orientation, no marked difference in enhancer activity was observed between clones of the two genotypes (mean value of 38.21 vs. 48.22) (*P* = 0.178) (Fig. 1B). Thus, genotype C2 has weaker ENI+ENII+CP activities but not weaker ENI+ENII activities than genotype B2, implicating a weaker CP in C2 than B2 genotype.

### Genotype C2 clones had weaker ENII and combined ENII+CP activities

**Figure 2:**
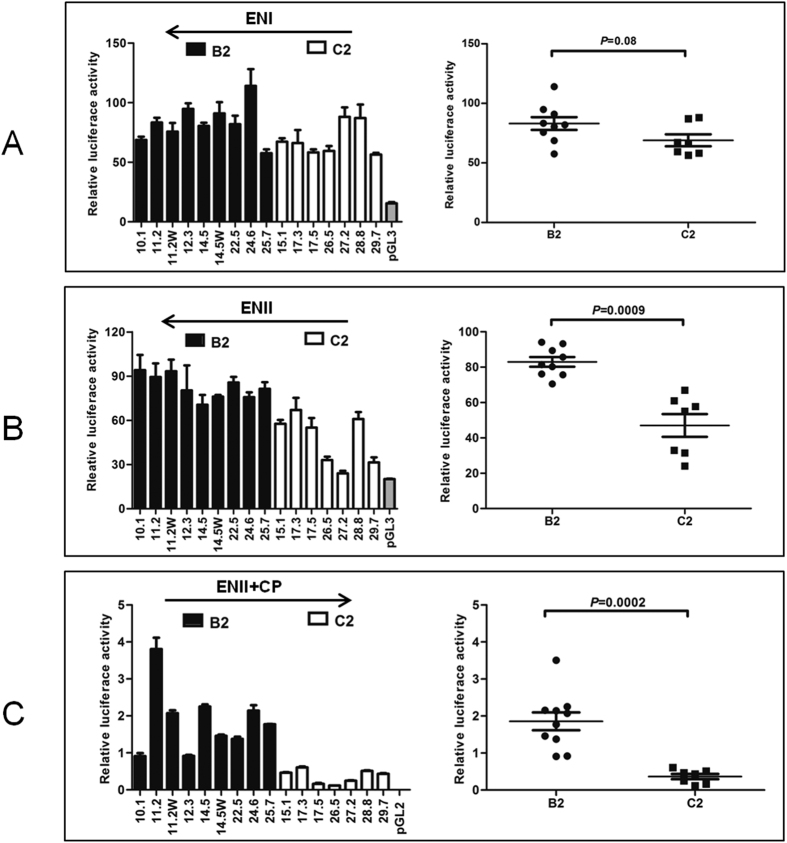
Comparison of activities of ENI (**A**), ENII (**B**), and ENII+CP (**C**) between HBV isolates of the B2 and C2 subgenotypes. ENI (873–1276) or ENII (1592–1780) was amplified from nine B2 clones and seven C2 clones, and subcloned into pGL3 promoter vector in the antisense orientation. Alternatively, ENII+CP (1627–1866) was amplified from these clones and subcloned to pGL2 Basic Vector in the sense orientation. The constructs were transfected to Huh7 cells and luciferase activity was measured from cell lysate two days later. Shown are results from three independent experiments. The relative luciferase levels (firefly luciferase activity/renilla luciferase activity) for individual clones are shown as columns in the left panels, and as dots in the right panel, with the P values indicated.

To more specifically compare ENI (873–1276) and ENII (1592–1780) activities between clones of the two genotypes, corresponding DNA fragments were subcloned to pGL3 Promoter vector in the antisense orientation. The seven C2 clones on average showed lower ENI activities than the nine B2 clones, but the difference did not reach statistical significance (*P* = 0.08; Fig. 2A). On the other hand, C2 clones had lower ENII activities than B2 clones with statistical significance (mean value: 47.01 vs. 82.96) (Fig. 2B).

To compare CP activities between the two genotypes, a DNA fragment covering position 1627–1866 was cloned to pGL2 Basic vector in the sense orientation. In transiently transfected Huh7 cells, C2 clones showed lower reporter activities than B2 clones (mean value: 0.36 vs. 1.86) (Fig. 2C). Similar results were obtained in HepG2 cells, another human hepatoma cell line (data not shown). Since ENII is embedded within CP, this result suggested that C2 clones had weaker CP and/or ENII activities.

### Four positions in the HBV genome partly accounted for genotype-specific difference in ENII+CP activities

**Figure 3:**
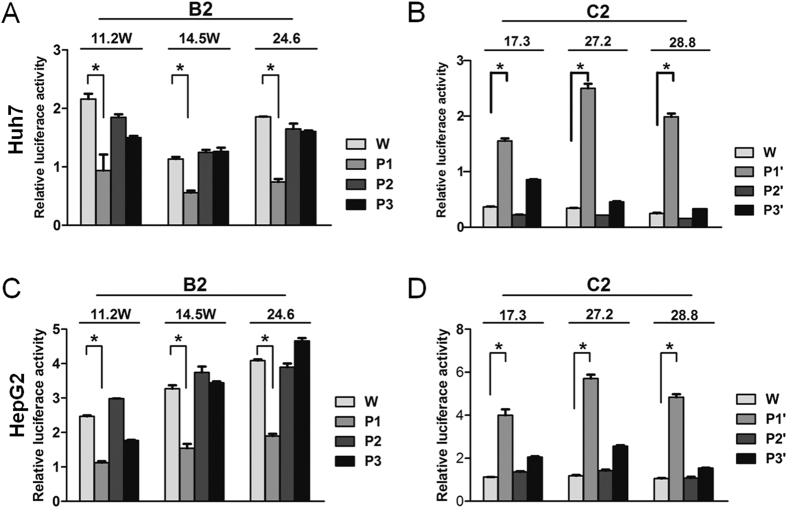
Impact of exchanging 5′ CP sequence between genotype B2 and C2 clones on ENII+CP activities. The ENII+CP reporter constructs of three B2 clones (**A**,**C**) and three C2 clones (**B**,**D**) were transfected to Huh7 cells (**A**,**B**) or HepG2 cells (**C**,**D**), and luciferase activities were measured 2 days later. W: original (WT) clone. P1, P2, and P3: converting B2 sequences at 1633/1635/1636/1652, 1673, and 1730 into C2 consensus sequences; P1′, P2′, and P3′: converting C2 sequences at these positions into B2 consensus sequences (see Table 1). The results were based on 3 repeat experiments. *P < 0.05.

To establish the molecular basis for differential ENII+CP activities, sequence alignment was performed on 453 isolates of genotype B and 525 isolates of genotype C available from GenBank (mostly of B2 and C2 subgenotypes). Seven positions within ENII+CP region showed genotype specific variations (Table 1), with most genotype B isolates having G1633, A1635, A1636, G1652, T1673, and G1730, in contrast to A1633, G1635, T1636, A1652, C1673, and C1730 found in isolates of genotype C. We also PCR amplified this region from the Chinese and US B2 and C2 isolates used in the previous study^18^, followed by sequencing analysis. All conformed to the dominant sequence in their respective genotype (data not shown). To determine the contribution of these divergent positions to different ENII+CP activities, site-directed mutagenesis was performed on three B2 clones and three C2 clones. Exchanging sequence at position 1673 or 1730 failed to significantly alter ENII+CP activity for either B2 or C2 clones in transiently transfected Huh7 cells (P2 and P3 in Fig. 3A; P2’ and P3′ in Fig. 3B). On the other hand, the P1 substitutions (G1633A/A1635G/A1636T/G1652A) in three B2 clones markedly reduced ENII+CP activities (Fig. 3A), whereas the P1′ substitutions (A1633G/G1635A/T1636A/A1652G) dramatically enhanced reporter activities for three C2 clones (Fig. 3B). Very similar results were obtained in transiently transfected HepG2 cells (Figs. 3C,D). Therefore, sequence divergence at these four positions at least partly accounted for different ENII+CP activities between the two genotypes.

### Exchanging ENII+CP overlapping region between clones of the two genotypes reversed the replication phenotype

**Figure 4:**
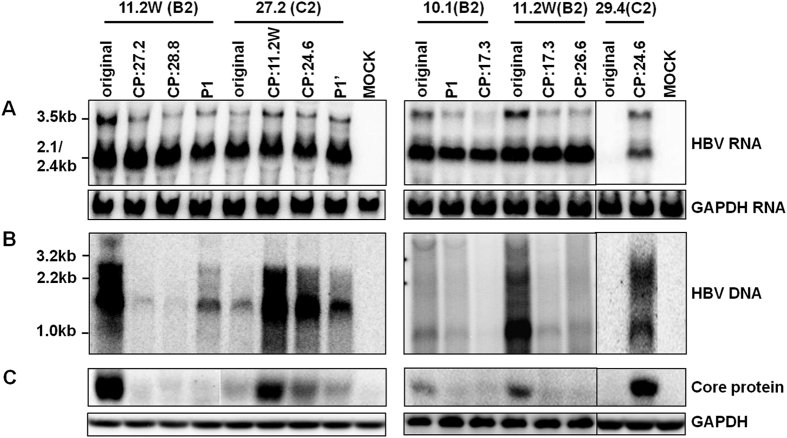
Impact of exchanging core promoter or just nucleotide positions 1633/1635/1636/1652 between B2 and C2 clones on HBV RNA transcription, DNA replication, and core protein expression. The name above the line indicates the original clone and their genotype. The name below indicates the source of core promoter. P1: G1633A/A1635G/A1636T/G1652A. P1′: A1633G/G1635A/T1636A/A1652G. CP: core promoter. Huh7 cells transiently transfected with dimeric HBV DNA constructs were harvested 3 days later. (**A**) Northern blot of HBV RNAs using GAPDH as a loading control. (**B**) Southern blot of replicative DNA; (**C**) Western blot of core protein with GAPDH serving as a loading control.

Results so far revealed weaker ENII, ENI+ENII+CP, and ENII+CP activities in C2 clones than B2 clones, with about 2-, 5-, and 5-fold differences in relative luciferase activities (Figs 1C and 2B,C). To establish the biological relevance of these findings, the ENII+CP overlapping region (1627–1866) was exchanged between clones of the two genotypes. Replacing ENII+CP of genotype B2 clone 11.2 W with those of genotype C2 clones (17.3, 26.6, 27.2 and 28.8) markedly reduced DNA replication (Fig. 4B). Conversely, ENII+CP from two genotype B2 clones (11.2 W and 24.6) substantially enhanced replication capacity of two C2 clones (27.2 and 29.4). Another genotype B2 clone (10.1) had relatively lower replication capacity than clone 11.2 W^18^. Replacement of its ENII+CP with that of clone 17.3 of genotype C further reduced DNA replication (Fig. 4B). The increase or reduction in replication capacity correlated with a corresponding change in the 3.5-kb RNAs (Fig. 4A) and core protein (Fig. 4C).

We also evaluated the impact of genotype-specific positions 1633, 1635, 1636, and 1652 on replication capacity. Introducing the P1 substitutions into the two genotype B2 clones (11.2 W and 10.1) reduced genome replication, albeit to a lesser extent than the swapping of the entire ENII+CP region (Fig. 4B). Conversely, the P1′ substitutions enhanced replication capacity of the C2 clone 27.2, but not as dramatically as replacement of ENII+CP.

## Discussion

We recently initiated comparative biological characterization of HBV genotypes B and C. Considering sequence variability of isolates belonging to the same genotype, we restricted our attention to subgenotypes B2 and C2 prevalent in China^18^. The full-length HBV genome was PCR amplified from a large number of serum samples from patients residing in China and United States, and converted to replication competent forms (circularized genome or vector-linked tandem dimer). This approach avoided the pitfall of making a conclusion based on a single “representative” clone. As core promoter mutations, which arise at the immune clearance phase of chronic infection, can augment HBV DNA replication^23^,^24^,^26^,^27^, isolates containing the well documented A1762T/G1764A mutations were excluded. Moreover, mixed probes of both subgenotypes were used during Southern/ Northern blot analyses and a mild washing condition (2XSSC/0.1% SDS at 65 °C) was adopted to ensure comparable detection sensitivity for different isolates of the same subgenotype.

With such an experimental design, we found more efficient DNA replication by the B2 isolates, but more efficient virion secretion by the C2 isolates^18^. The higher replication capacity of the B2 isolates correlated with higher level of the 3.5-kb RNA, which drives HBV DNA replication by serving not only as the genome precursor, but also as mRNA for both core and P proteins. As the core protein is a strong immunogen, we propose that the high replication capacity of genotype B2 will trigger earlier immune attack (shorten the immune tolerance phase) and accelerate HBeAg seroconversion^4^,^5^,^6^. This will diminish the lifelong risk for liver cirrhosis and HCC^7^,^8^,^9^,^10^ but cause early HCC development unaccompanied by liver cirrhosis^9^. The strong immune response could also increase the risk for fulminant hepatitis during acute adulthood infection but reduce the chronicity rate^15^,^16^,^17^,^18^. It could also explain why genotype B patients are more responsive to interferon therapy than genotype C patients^11^,^12^. On the other hand, the low replication capacity of wild-type genotype C isolates may serve as a driving force for the emergence of core promoter mutations during the immune clearance phase of infection. Indeed, genotype C isolates are more likely to develop A1762T/G1764A and other core promoter mutations than genotype B isolates^6^,^28^,^29^, which augment genome replication by transcriptional up regulation of pgRNA at the expense of pcRNA^23^,^24^,^26^ and are an independent risk factor for HCC development^30^,^31^,^32^. Detailed immunological studies are needed to verify our hypothesis.

The objective of the present study was to clarify why genotype B2 isolates with wild-type core promoter sequence have higher replication capacity than genotype C2 isolates. While this can be achieved by systemic exchange of genomic fragments between clones of the two genotypes, an alternative approach is to compare transcriptional regulatory elements for the pg RNA. In this regard transcription of the 3.5-kb RNA is driven by the core promoter. In addition, the two enhancer elements further augment HBV RNA transcription^19^,^20^,^21^. By inserting a DNA fragment (873–1866) covering both ENI and ENII into a reporter vector in the antisense orientation, we failed to observe significant difference in the combined ENI+ENII activities between clones of subgenotypes B2 and C2 (Fig. 1B). When tested separately, however, subgenotype C2 had weaker ENII activity than subgenotype B2 (Fig. 2B). ENI (873–1276) from the C2 isolates also appeared less active, although the difference with B2 isolates did not reach statistical significance (Fig. 2A). A possible explanation for the apparently contradictory findings is that the intervening sequence between the two enhancers (1277–1591) negatively regulates enhancer activity, with the inhibitory effect being greater for genotype B2. Indeed, a negative regulatory element (NRE) of transcription has been mapped to positions 1455–1626 (Fig. 1A)^33^,^34^.

A large portion of the core promoter is overlapped by enhancer II, making it difficult to establish the relative contribution of CP vs. ENII to the reporter activity observed. Still, several pieces of evidence suggest that genotype B2 has a stronger CP than genotype C2. First, when the 1-kb DNA fragment covering ENI+ENII+CP (and also NRE) was inserted to a promoter vector in the antisense orientation, the mean enhancer activity was similar between the two genotypes (45 vs. 40) (Fig. 1B). When the same DNA fragment was inserted to a promoterless vector in a sense orientation, the reporter activity was about 5 times stronger for B2 clones than C2 clones (Fig. 1C). Second, genotype B2 clones displayed about 1.8-fold higher ENII activity than C2 clones (Fig. 2B) but 5-fold higher ENII+CP activity than C2 clones (Fig. 2C). Further studies revealed that genotype-specific sequence variability at positions 1633, 1635, 1636, and 1652 is partly responsible for different promoter/enhancer activity (Fig. 3). Finally, exchanging the ENII+CP overlapping region, and to a lesser extent, nucleotide positions 1633, 1635, 1636, and 1652, reversed replication capacity of several genotype B2 and C2 clones (Fig. 4). This last finding demonstrated that the higher replication capacity of genotype B2 isolates could be explained, at least in part, by a more active ENII+CP region.

A drawback of the current study is that only the tandem dimer was used for transfection experiment. During authentic HBV infection, the 3.2-kb covalently closed circular (ccc) DNA serves as the template for HBV RNA transcription^35^. The cccDNA forms minichromosome, with transcription regulated by histone modifications. Circularity of the cccDNA is disrupted by vector sequence in the tandem SphI dimer. It will be interesting to validate the major findings using linearized or recircularized HBV genome to mimic cccDNA^36^,^37^. Alternative, Cre/loxP-mediated DNA recombination can be used to generate a cccDNA-like molecule inside transfected cells^38^.

## Additional Information

**How to cite this article**: Qin, Y. *et al.* Stronger enhancer II/core promoter activities of hepatitis B virus isolates of B2 subgenotype than those of C2 subgenotype. *Sci. Rep.* **6**, 30374; doi: 10.1038/srep30374 (2016).

## Supplementary Material

Supplementary Information

## Figures and Tables

**Table 1:** Divergent nucleotide positions in the ENII+CP region between genotype B and C.

	Genotype B*				Genotype C**			
	**G**	**A**	**C**	**T**	**G**	**A**	**C**	**T**
**nt1633**	**0.945**	0.049	0.004	0.002	0.013	**0.973**	0.001	0.013
**nt1635**	0.046	**0.945**	0.007	0.002	**0.99**	0	0	0.01
**nt1636**	0.004	**0.936**	0.004	0.042	0.009	0.011	0.03	**0.95**
**nt1652**	**0.817**	0.181	0.002	0	0.023	**0.977**	0	0
**nt1673**	0.02	0.002	0.073	**0.905**	0.002	0	**0.983**	0.015
**nt1730**	**0.923**	0.011	0.066	0	0.021	0.002	**0.977**	0

^*^453 isolates.

^**^525 isolates.

## References

[b1] Chu C. J. & Lok A. S. F. Clinical significance of hepatitis B virus genotypes. Hepatology 35, 1274–1276, doi: 10.1053/jhep.2002.33161 (2002). 11981779

[b2] Norder H. *et al.* Genetic diversity of hepatitis B virus strains derived worldwide: Genotypes, subgenotypes, and HBsAg subtypes. Intervirology 47, 289–309, doi: 10.1159/000080872 (2004). 15564741

[b3] Okamoto H. *et al.* Typing Hepatitis-B Virus by Homology In Nucleotide-Sequence - Comparison Of Surface-Antigen Subtypes. J Gen Virol 69, 2575–2583, doi: 10.1099/0022-1317-69-10-2575 (1988). 3171552

[b4] Chu C. J., Hussain M. & Lok A. S. F. Hepatitis B virus genotype B is associated with earlier HBeAg seroconversion compared with hepatitis B virus genotype C. Gastroenterology 122, 1756–1762, doi: 10.1053/gast.2002.33588 (2002). 12055581

[b5] Orito E. *et al.* Geographic distribution of hepatitis B virus (HBV) genotype in patients with chronic HBV infection in Japan. Hepatology 34, 609a–609a (2001). 10.1053/jhep.2001.27221 11526547

[b6] Yuen M. F. *et al.* Significance of hepatitis B genotype in acute exacerbation, HBeAg seroconversion, cirrhosis-related complications, and hepatocellular carcinoma. Hepatology 37, 562–567, doi: 10.1053/jhep.2003.50098 (2003). 12601354

[b7] Chan H. L. Y. *et al.* Genotype C hepatitis B virus infection is associated with an increased risk of hepatocellular carcinoma. Gut 53, 1494–1498, doi: 10.1136/gut.2003.033324 (2004). 15361502 PMC1774221

[b8] Chu C. M. & Liaw Y. F. Genotype C hepatitis B virus infection is associated with a higher risk of reactivation of hepatitis B and progression to cirrhosis than genotype B: A longitudinal study of hepatitis B e antigen-positive patients with normal aminotransferase levels at baseline. Journal of hepatology 43, 411–417, doi: 10.1016/j.jhep.2005.03.018 (2005). 16006001

[b9] Kao J. H., Chen P. J., Lai M. Y. & Chen D. S. Hepatitis B genotypes correlate with clinical outcomes in patients with chronic hepatitis B. Gastroenterology 118, 554–559 (2000). 10702206 10.1016/s0016-5085(00)70261-7

[b10] Sumi H. *et al.* Influence of hepatitis B virus genotypes on the progression of chronic type B liver disease. Hepatology 37, 19–26, doi: 10.1053/jhep.2003.50036 (2003). 12500184

[b11] Kao J. H., Wu N. H., Chen P. J., Lai M. Y. & Chen D. S. Hepatitis B genotypes and the response to interferon therapy. Journal of hepatology 33, 998–1002 (2000). 11131465 10.1016/s0168-8278(00)80135-x

[b12] Wai C. T., Chu C. J., Hussain M. & Lok A. S. F. HBV genotype B is associated with better response to interferon therapy in HBeAg(+) chronic hepatitis than genotype C. Hepatology 36, 1425–1430, doi: 10.1053/jhep.2002.37139 (2002). 12447868

[b13] Wen W. H. *et al.* Secular trend of the viral genotype distribution in children with chronic hepatitis B virus infection after universal infant immunization. Hepatology 53, 429–436, doi: 10.1002/hep.24061 (2011). 21274864

[b14] Zhang H. W. *et al.* Risk factors for acute hepatitis B and its progression to chronic hepatitis in Shanghai, China. Gut 57, 1713–1720, doi: 10.1136/gut.2008.157149 (2008). 18755887 PMC2582333

[b15] Imamura T. *et al.* Distribution of hepatitis B viral genotypes and mutations in the core promoter and precore regions in acute forms of liver disease in patients from Chiba, Japan. Gut 52, 1630–1637, doi: 10.1136/gut.52.11.1630 (2003). 14570734 PMC1773865

[b16] Ozasa A. *et al.* Influence of genotypes and precore mutations on fulminant or chronic outcome of acute hepatitis B virus infection. Hepatology 44, 326–334, doi: 10.1002/Hep.21249 (2006). 16871568

[b17] Ren X. *et al.* Hepatitis B virus genotype and basal core promoter/precore mutations are associated with hepatitis B-related acute-on-chronic liver failure without pre-existing liver cirrhosis. Journal of viral hepatitis 17, 887–895, doi: 10.1111/j.1365-2893.2009.01254.x (2010). 20070500 PMC2998700

[b18] Qin Y. L. *et al.* Hepatitis B Virus Genotype C Isolates with Wild-Type Core Promoter Sequence Replicate Less Efficiently than Genotype B Isolates but Possess Higher Virion Secretion Capacity. J Virol 85, 10167–10177, doi: 10.1128/Jvi.00819-11 (2011). 21775451 PMC3196399

[b19] Shaul Y., Rutter W. J. & Laub O. A Human Hepatitis-B Viral Enhancer Element. Embo J 4, 427–430 (1985). 3926485 10.1002/j.1460-2075.1985.tb03646.x PMC554203

[b20] Wang Y. *et al.* A New Enhancer Element, Enii, Identified In the X-Gene Of Hepatitis-B Virus. J Virol 64, 3977–3981 (1990). 2370684 10.1128/jvi.64.8.3977-3981.1990 PMC249695

[b21] Yuh C. H. & Ting L. P. The genome of hepatitis B virus contains a second enhancer: cooperation of two elements within this enhancer is required for its function. J Virol 64, 4281–4287 (1990). 2166817 10.1128/jvi.64.9.4281-4287.1990 PMC247894

[b22] Zong L. *et al.* Two-way molecular ligation for efficient conversion of monomeric hepatitis B virus DNA constructs into tandem dimers. Journal of virological methods 233, 46–50, doi: 10.1016/j.jviromet.2016.03.012 (2016). 27025357 PMC5257262

[b23] Parekh S. *et al.* Genome replication, virion secretion, and e antigen expression of naturally occurring hepatitis B virus core promoter mutants. J Virol 77, 6601–6612, doi: 10.1128/Jvi.77.12.6601-6612.2003 (2003). 12767980 PMC156182

[b24] Tsai A. *et al.* Chimeric constructs between two hepatitis B virus genomes confirm transcriptional impact of core promoter mutations and reveal multiple effects of core gene mutations. Virology 387, 364–372, doi: 10.1016/j.virol.2009.03.002 (2009). 19327810 PMC2893023

[b25] Guo H., Mao R., Block T. M. & Guo J. T. Production and function of the cytoplasmic deproteinized relaxed circular DNA of hepadnaviruses. J Virol 84, 387–396, doi: 10.1128/JVI.01921-09 (2010). 19864387 PMC2798433

[b26] Buckwold V. E., Xu Z. C., Chen M., Yen T. S. B. & Ou J. H. Effects of a naturally occurring mutation in the hepatitis B virus basal core promoter on precore gene expression and viral replication. J Virol 70, 5845–5851 (1996). 8709203 10.1128/jvi.70.9.5845-5851.1996 PMC190601

[b27] Jammeh S., Tavner F., Watson R., Thomas H. C. & Karayiannis P. Effect of basal core promoter and pre-core mutations on hepatitis B virus replication. J Gen Virol 89, 901–909, doi: 10.1099/vir.0.83468-0 (2008). 18343830

[b28] Orito E. *et al.* A case-control study for clinical and molecular biological differences between hepatitis B viruses of genotypes B and C. Japan HBV Genotype Research Group. Hepatology 33, 218–223, doi: 10.1053/jhep.2001.20532 (2001). 11124839

[b29] Wang Z. H. *et al.* Clinical and virological characteristics of hepatitis B virus subgenotypes Ba, C1, and C2 in China. J Clin Microbiol 45, 1491–1496, doi: 10.1128/Jcm.02157-06 (2007). 17376881 PMC1865908

[b30] Baptista M., Kramvis A. & Kew M. C. High prevalence of 1762(T) 1764(A) mutations in the basic core promoter of hepatitis B virus isolated from black Africans with hepatocellular carcinoma compared with asymptomatic carriers. Hepatology 29, 946–953, doi: 10.1002/hep.510290336 (1999). 10051502

[b31] Kao J. H., Chen P. J., Lai M. Y. & Chen D. S. Basal core promoter mutations of hepatitis B virus increase the risk of hepatocellular carcinoma in hepatitis B carriers. Gastroenterology 124, 327–334, doi: 10.1053/gast.2003.50053 (2003). 12557138

[b32] Kuang S. Y. *et al.* Specific mutations of hepatitis B virus in plasma predict liver cancer development. Proc Natl Acad Sci USA 101, 3575–3580, doi: 10.1073/pnas.0308232100 (2004). 14990795 PMC373504

[b33] Chen M. & Ou J. H. Cell type-dependent regulation of the activity of the negative regulatory element of the hepatitis B virus core promoter. Virology 214, 198–206, doi: 10.1006/viro.1995.9940 (1995). 8525615

[b34] Lo W. Y. & Ting L. P. Repression of enhancer II activity by a negative regulatory element in the hepatitis B virus genome. J Virol 68, 1758–1764 (1994). 8107237 10.1128/jvi.68.3.1758-1764.1994 PMC236636

[b35] Guo J. T. & Guo H. Metabolism and function of hepatitis B virus cccDNA: Implications for the development of cccDNA-targeting antiviral therapeutics. Antiviral research 122, 91–100, doi: 10.1016/j.antiviral.2015.08.005 (2015). 26272257 PMC4586118

[b36] Gunther S. *et al.* A novel method for efficient amplification of whole hepatitis B virus genomes permits rapid functional analysis and reveals deletion mutants in immunosuppressed patients. J Virol 69, 5437–5444 (1995). 7636989 10.1128/jvi.69.9.5437-5444.1995 PMC189390

[b37] Qin Y. *et al.* Improved method for rapid and efficient determination of genome replication and protein expression of clinical hepatitis B virus isolates. J Clin Microbiol 49, 1226–1233, doi: 10.1128/JCM.02340-10 (2011). 21289153 PMC3122861

[b38] Qi Z. *et al.* Recombinant covalently closed circular hepatitis B virus DNA induces prolonged viral persistence in immunocompetent mice. J Virol 88, 8045–8056, doi: 10.1128/JVI.01024-14 (2014). 24807718 PMC4097776

