# Supplementary material for: Stronger enhancer II/core promoter activities of hepatitis B virus isolates of B2 subgenotype than those of C2 subgenotype
Source: Sci Rep. 2016 Jul 27;6:30374. doi: 10.1038/srep30374 (PMC4961966; doi:10.1038/srep30374)
Supplement: Supplementary Information [file srep30374-s1.pdf]

Stronger enhancer II/core promoter activities of hepatitis B virus isolates of B2 subgenotype than those of C2 subgenotype

Yanli Qin[1], Xueshi Zhou[3], Haodi Jia[4], Chaoyang Chen[4], Weifeng Zhao[3], Jiming Zhang[1]# and Shuping Tong[2, 4]#

[1]Department of Infectious Diseases, Huashan Hospital, Fudan University, Shanghai, China; [2]The Liver Research Center, Rhode Island Hospital, Warren Alpert School of Medicine, Brown University, Providence, RI, USA; [3]Department of Infectious Diseases, the First Affiliated Hospital of Soochow University, Suzhou, Jiangsu Province, China; [4]Key Lab of Medical Molecular Virology, School of Basic Medical Sciences, Fudan University, Shanghai, China.

Running head: Core promoter activities of HBV B2 vs. C2 genotype

# Address correspondence to Jiming Zhang, M.D, Department of Infectious Diseases, Huashan Hospital, 12 Middle Wulumuqi Road, Shanghai 200040, China. Phone: +86 21 52888125. Fax: +86 21 62486140. Email: jmzhang@fudan.edu.cn; Shuping Tong, M.D.; Ph.D. Liver Research Center, Rhode Island Hospital, 55 Claverick Street, Providence, RI 02903, USA. Phone: 01-401-444-7365. Fax: 01-401-444-2939. Email: shuping_tong_md@brown.edu.

Y.Q. and X.Z. contributed equally to this work.

Supplementary Table. 1. Primers used to amplify HBV transcriptional regulatory elements and restriction sites used for cloning of PCR products.

Regulatory elements	Primer	Sequence (5'-3')*	Position	Restrition site
ENI+ENII	S1-a	TCGAT***C TCGAG***CTTCATGGGATATGTAATTGGGGGTTG	876-898	XhoI
	S1-b	TCGAT***C TCGAG***CTTCATGGGATATGTAATTGGAAG	876-898	
	S1-c	TCGAT***C TCGAG***CTTCATGGGATATGTAATTGGGAG	876-898	
	AS1	CACCCAAGGCACAGCTTGG***G AGCTC***GAACAGT	1857-1886	SacI
ENI+ENII+CP	S2-a	TCGAT***GAGCT C***CTTCATGGGATATGTAATTGGGGGTTG	873-899	SacI
	S2-b	TCGAT***GAGCT C***CTTCATGGGATATGTAATTGGAAG	873-896	
	S2-c	TCGAT***GAGCT C***CTTCATGGGATATGTAATTGGGAG	873-896	
	AS2	CACCCAAGGCACAGCTTGG***A AGCTT***GAACAGT	1871-1886	HindIII
ENI	S3-a	TCGAT***A GATCT***CTTCATGGGATATGTAATTGGGGGTTG	873-899	BglII
	S3-b	TCGAT***A GATCT***CTTCATGGGATATGTAATTGGAAG	873-896	
	S3-c	TCGAT***A GATCT***CTTCATGGGATATGTAATTGGGAG	873-896	
	AS3	TATT***GAGCT C***GCAGTATGGATCGGCAGAGGAG	1276-1253	SacI
ENII	S4	CACT***C TCGAG***CCTCTGCACGTCGCATGGA	1584-1612	XhoI
	AS4	GACCAATTTATGCCT***GGTAC C***TCC	1798-1776	KpnI
ENII+CP	S5	GAAACCACCGT***GAGCT C***CCACC	1611-1632	SacI
	AS5	CAAGGCACAGCTTGG***A AGCTT***GAAC	1886-1862	HindIII

*underlined sequences are HBV specific. Restriction sites are in bold.
